# Water-soluble dye staining of the flap nutrient artery and its perforator branch in cadavers embalmed using saturated salt solution and urea methods: does demonstration using a dye-stained cadaver increase a trainee’s level of skill acquisition?

**DOI:** 10.1007/s12565-025-00823-8

**Published:** 2025-02-02

**Authors:** Yutaro Araki, Yuzuka Oda, Mikitaka Kitagawa, Kohei Aoki, Takako Komiya, Mamoru Kikuchi, Takayuki Shirai, Shinichi Kawata, Masahiro Itoh, Hajime Matsumura

**Affiliations:** 1 Department of Plastic and Reconstructive Surgery, Tokyo Medical University, 6-7-1 Nishi-Shinjuku, Shinjuku-ku, Tokyo, 160-0023 Japan; 2 Shimokitazawa Hospital, Tokyo, Japan; 3 Department of Plastic and Reconstructive Surgery, Niizashiki Central General Hospital, Tokyo, Japan; 4 Department of Anatomy, Tokyo Medical University, Tokyo, Japan

**Keywords:** Cadaver, Formaldehyde, Iodine, Staining and labeling, Urea

## Abstract

Cadaveric surgical training (CST) can safely improve surgical skill. Recently, various innovations have made surgical training more realistic. Saturated salt solution (SSS) and urea methods are useful for plastic surgery training, because they better preserve the color and texture of soft tissues than formalin fixation (FA). Microvessels are invisible to the naked eye, and in this study, we visualized skin perforators by injecting dye into the flaps of nutrient vessels during hand and foot surgery. During each CST session, the instructor used three cadavers for demonstration. From 2017 to 2021, these cadavers were not stained, whereas from 2022 to 2023, the cadavers were stained to enhance the visualization of nutrient and perforating vessels. We compared the self-rated skill gains of participants who observed demonstrations on unstained cadavers from 2017 to 2021 and participants who observed demonstrations on stained cadavers from 2022 to 2023. Among 36 participants from the 2022–2023 group, 28 rated the staining of nutrient vessels and perforating branches as adequate. Of 32 participants, 29 preferred the use of dyed cadavers during training. Trainee skill increase for the digital artery flap, reverse digital artery flap, and dorsal metacarpal artery perforator flap was significantly higher in 2022–2023 (with stained cadavers) than in the earlier course without stained cadavers. SSS-fixed and urea-treated cadavers combined with vascular staining may be useful training models for flap elevation.

## Introduction

Surgical technique training using cadaver surgical training (CST) can safely improve surgical skills. The hands-on use of cadavers allows surgeons to understand human anatomy in three dimensions and simulate surgery. This bridges the gap between theoretical knowledge and surgical practice. In recent years, various innovations have been made to make training more realistic.

The light embalming method is valuable for surgical training, as it preserves the color and texture of fresh tissue to closely resemble that of a living body and reduces health risks for trainees compared to traditional high-formalin methods (Anderson et al. 2006). Therefore, efforts to reduce formalin concentrations during cadaver preservation are becoming important in surgical training. Jaung et al. (2011) discovered that altering the composition of the solution used for embalming improves color tone and joint range of motion.

Since 2017, CST seminars have been organizing CST seminars as university research projects. One of the features of our CST is the use of a saturated salt solution (SSS) and urea in cadavers. The SSS method uses SSS as the fixative (Hayashi et al. 2014). The urea method neutralizes formalin by irrigation with urea solution, which is a scavenger, after conventional formaldehyde fixation (Otsuka et al. 2022). Formalin-fixed catheters differ significantly from living soft tissues in terms of texture and color (Hassan et al. 2015). The SSS and urea methods have been reported to be useful for training in plastic surgery, because, compared to conventional formalin fixation, the color and texture of cadaver soft tissues are more similar to those of living tissues (Shirai et al. 2022). However, because minute blood vessels, such as the skin perforators, are invisible to the naked eye, perforator branch preservation cannot be confirmed during training for perforator flap elevation.

This study aimed to confirm whether SSS-fixed and urea-treated cadavers can stain the skin flap nutrient artery and its perforator, which are commonly used in plastic surgery. Instructors then used a stained cadaver to demonstrate skin flap elevation at the CST. We then examined whether trainees’ skills could be improved using an artery-stained cadaver.

## Materials and methods

### Cadaver preparation and fixation

#### SSS fixation

Each cadaver was fixed using a solution consisting of sodium chloride (20 kg), 20% formaldehyde (1 L), phenol (0.2 L), glycerin (0.5 L), isopropyl alcohol (4.0 L), and water (19.3 L). The solution was injected through the femoral artery.

#### *FA* + *urea fixation*

FA consisted of 20% formaldehyde (3 L), phenol (0.5 L), and water (6.5 L). Each cadaver was treated with FA, followed by reperfusion with a solution consisting of 60% alcohol (18 L), phenol (0.2 L), glycerin (0.2 L), urea (1 kg), and water (1.5 L). The solution was injected through the femoral artery.

### Dye injection procedure

The staining solution was a mixture of 2 g of water-based acrylic paint (“Efu Watercolor Paints WFCT15, Pink,” PENTEL, Tokyo, Japan), 10 mL of iodine tincture (“TY” Taiyo Pharmaceutical, Tokyo, Japan), and 40 mL water. The solution was injected immediately before the instructor’s demonstration. A 5–7 Fr pediatric urinary catheter, depending on the size of the nutrient vessel, was introduced into the artery. The cannulation site was double-ligated with a 4–0 nylon thread to prevent leakage.

### Cadaver surgical training (CST) setup

Our CST, which has been held since 2017 in cooperation with the Department of Anatomy, consisted of a 1-day program each for hand and foot surgery. The contents of the hand surgery program included basic hand surgery techniques (tendon suture, nerve dissection, carpal tunnel release, tendon sheath incision, etc.), local skin flaps for the hand (digital artery flap, reverse digital artery flap, dorsal metacarpal artery perforator flap, etc.), and radial forearm skin flaps. The foot surgery program included Achilles tendon lengthening, gastrocnemius fasciotomy (using an endoscope), flexor tendonotomy, metatarsal osteotomy, anterolateral thigh skin flap (free choice), fibular osteocutaneous flap (free choice), and medial plantar flap (free choice).

After the lecture and demonstration by the instructor, each trainee demonstrated their acquired skills. The program consisted of approximately 1 h of lectures by the instructor, 1 h of demonstrations, and about 5 h of hands-on practice. For the hand surgery course, one limb was provided per two participants, while in the foot surgery course, one limb was provided per participant. Participants were asked to self-assess their confidence levels (SACL score) before and after the training, and the rate of increase in proficiency before and after training was quantitatively evaluated.

This study was approved by the Ethics Committee of Tokyo Medical University (ethics review numbers: T2019-0125 and SH3809). Informed consent was obtained either from the individuals before death or from their families after death. The authors confirm that every effort was made to comply with all local and international ethical guidelines and laws concerning the use of human cadaveric donors in anatomical research.

Three cadavers were stained for demonstrations by CST instructors: two on November 6, 2022, and October 29, 2023 (hand surgery course), and one on January 22, 2023 (foot surgery course). The nutrient arteries and their perforating branches were stained with dye in the flaps of the forearm, digital artery, reverse digital artery, dorsal metacarpal artery perforator, anterolateral thigh-free, fibular osteocutaneous, and medial plantar regions. Apart from the staining of vessels, the program contents in the CST from 2017 to 2021 were identical to those of 2022–2023.

Participants were recruited via the university’s website, email, and social networking sites. Physicians in plastic or orthopedic surgery beyond their third year of medical licensure were considered. There were no specific exclusion criteria, but preference was given to those with specialist certifications. The instructors for all seminars were consistent, comprising one university staff member and three non-staff members.

The survey was administered to 32 participants with 3–17 years of experience in plastic surgery (*n* = 17) and orthopedic surgery (*n* = 15), as well as to three instructors of the hand surgery course held in 2022–2023. The questions on the survey included items regarding the degree of staining, desire to stain their own cadavers, and the SACL score. A survey of instructors was conducted only for the hand surgery course in 2022, and the survey questions were limited to items regarding the degree of staining. The number of years after obtaining a medical license was 5.19 ± 1.75 and 6.56 ± 4.05 years for participants in 2022 and 2023, respectively. The SACL score was measured not only for participants in 2022–2023 but also for those from 2017 to 2021 (no event was held in 2020 because of the COVID-19 pandemic). From 2017 to 2021, the average number of years since obtaining a medical license was 6.13 ± 2.88 years in 2017, 6.94 ± 2.46 years in 2018, 5.79 ± 3.53 years in 2019, and 7.55 ± 2.76 years in 2021. During this period, 49 and 48 participants were from plastic and orthopedic surgery specialties, respectively.

### Evaluation of outcomes

#### Staining of vessels

In 2022 and 2023, a questionnaire was administered to both participants of the hand surgery courses in 2022 and 2023, as well as to the instructors of the 2022 hand surgery course, to evaluate the adequacy of the cadaver vascular staining. Staining adequacy was rated on a 5-point scale: Excessive, Slightly Excessive, Moderate, Slightly Insufficient, and Insufficient. Additionally, participants in the hand surgery courses in 2022 and 2023 were asked whether they would prefer the practice cadaver to be stained like the demonstration cadaver. This preference was rated on a 4-point scale: Preferred, Relatively Preferred, Relatively Unpreferred, and Unpreferred.

#### Educational effectiveness

Hand surgery trainees from 2017 to 2023 were asked to self-assess their confidence level (SACL score) before and after training. The scoring system was set as follows: 0 indicated no confidence in completing the surgery, 5 indicated confidence in completing the surgery if an experienced assistant was present, and 10 indicated confidence in completing the surgery even if a resident was assisting. The difference between the pre- and post-training scores was calculated to determine the increase in trainees’ skill acquisition. To assess the impact of staining demonstration cadavers on participants’ anatomical understanding, the SACL scores of participants from 2022 to 2023 (who used cadavers with stained vasculature) were compared to those from 2017 to 2021 (who used non-stained cadavers) in hand surgery skills related to flap elevation. As flap elevation was not included in the foot surgery course, foot surgery was not evaluated.

All questionnaires were created using Google Forms (Google, California, USA) and were conducted non-anonymously. For the pre-training SACL score, the questionnaire was distributed 2 weeks before the training, and responses were collected before the start of the training. Responses for other items were collected immediately after the training.

### Statistical analysis

Data were analyzed using JMC17 for Mac. Means and standard deviations were calculated to summarize the increase in SACL scores for each flap type in the unstained (2017–2021) and dye-stained (2022–2023) groups. Independent sample t tests were used to compare the means between the unstained (2017–2021) and dye-stained (2022–2023) groups, and the results were considered statistically significant at p < 0.05.

## Results

### Results of staining nutrient vessels and their perforating branches

**Fig. 1:**
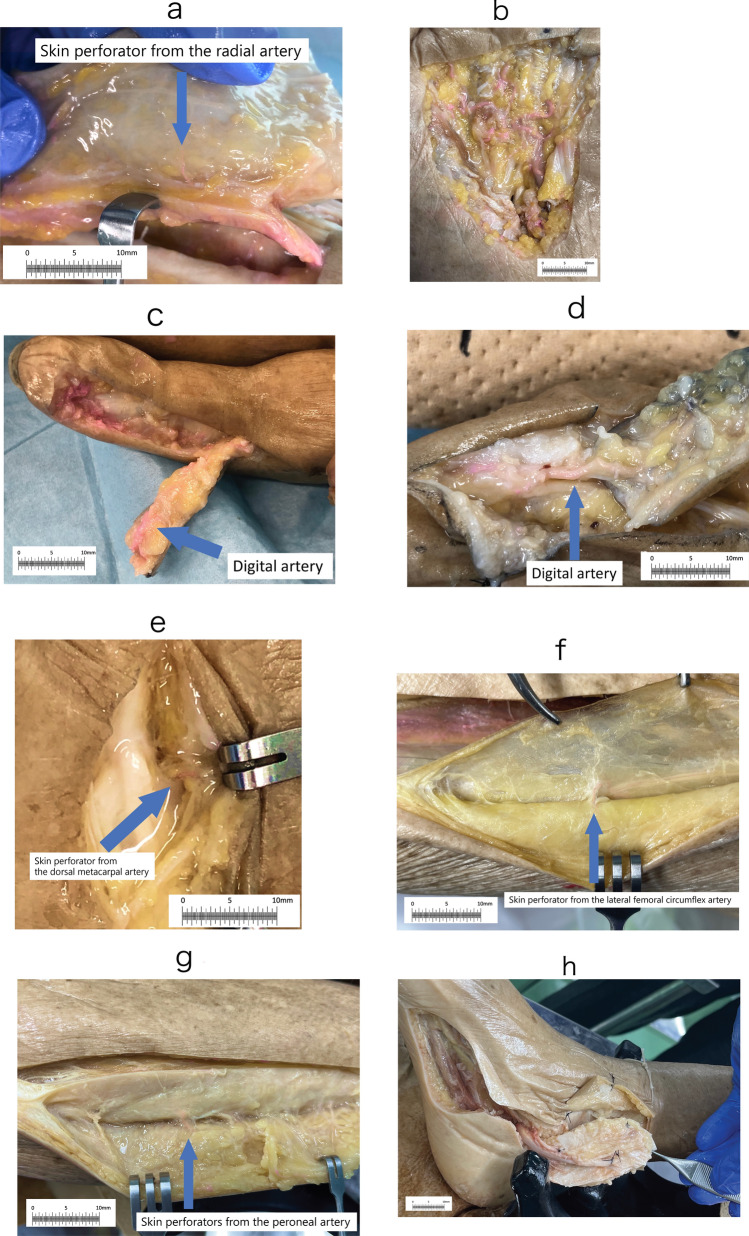
Dye-stained nutrient vessels and perforating branches for each skin flap. Except for the medial plantar flap, the nutrient vessels and their perforating branches were well stained. **a** Forearm flap. Fixation method: SSS method; Cannulation: Brachial artery; Injection volume: 8 cc. The blue arrow shows a skin perforator from the radial artery. **b** Palmar superficial artery arch. Fixation method: SSS method; Cannulation: Radial artery; Injection volume: 5 cc. **c** Digital artery flap. Fixation method: SSS method; Cannulation: Radial artery; Injection volume: 5 cc. **d** Retrograde digital artery flap. Fixation method: SSS method; Cannulation: Radial artery; Injection volume: 5 cc. **e** Dorsal metacarpal artery perforation branch flap. Fixation method: SSS method; Cannulation: Radial artery; Injection volume: 5 cc. **f** Anterolateral femoral flap. Fixation method: Urea method; Cannulation: Lateral femoral circumflex descending branch; Injection volume: 3 cc. **g** Fibular flap Fixation method: Urea method; Cannulation: Popliteal artery; Injection volume: 5 cc. **h** Medial plantar flap Cannulation: Posterior tibial artery; cannulation was difficult due to severe calcification of the posterior tibial artery

Figure 1 shows the amount of staining solution injected and the staining of the nutrient vessels and perforating branches for each skin flap type. Except for the medial plantar flap, the nutrient vessels and their perforating branches were well stained. In the medial plantar flap, the posterior tibial artery itself was highly calcified, and cannulation was impossible.

In the digital artery flap, when the digital artery was cut off at its distal end, the dye leaked into the soft tissue distal to the skin flap elevation.

### Evaluation of staining from the participant’s point of view

The response rate for all questions for each year and item was 100%. Twenty-eight of the 36 participants, including the instructor, rated the staining of the nutrient vessels and perforating branches as adequate, five rated it as excessive, and none rated it as insufficient. Twenty-nine of the 32 participants answered that they preferred dyeing during training (Table 1).

**Table 1:** Evaluation of the water-soluble dye staining from the participant’s point of view (2022 − 2023)

	Excessive	Slightly excessive	Adequate	Slightly insufficient	Insufficient
Evaluation of staining (*n* = 36)	0	5	28	3	0
(%)	0	13.9	77.8	8.3	0

	Preferred	Relatively preferred	Relatively unpreferred	Not preferred	
Preference for dyeing (*n* = 32)	22	7	2	1	
(%)	68.8	21.9	6.3	3.1	

### Comparison of the increase in skill acquisition with/without dye staining

**Fig. 2:**
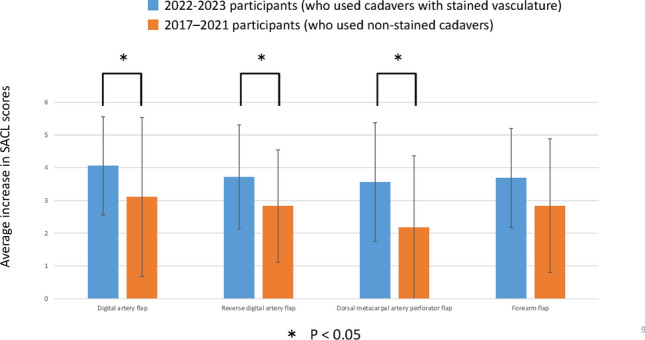
Comparison of the increase in skill acquisition with/without the dye-stained demonstration. The increase in trainee skill for the digital artery flap, reverse digital artery flap, and dorsal metacarpal artery perforator flap was significantly higher in 2022–2023 (with stained cadavers) than in the earlier course without stained cadavers

The response rate for all questions in each year and item was 100%. For the digital artery flap, trainees in the 2022–2023 course with dye staining exhibited a significantly higher average increase in the SACL score (M = 4.06, SD = 1.50) than trainees in the 2017–2021 courses without stained cadavers (M = 3.11, SD = 2.42). Similar improvements were observed for the reverse digital artery flap (2022–2023: M = 3.72, SD = 1.59 vs. 2017–2021, M = 2.83, SD = 1.71), dorsal metacarpal artery perforator flap (2022–2023: M = 3.56, SD = 1.82 vs. 2017–2021: M = 2.18, SD = 2.19), and forearm flap (2022–2023: M = 3.69, SD = 1.51 vs. 2017–2021: M = 2.84, SD = 2.04), with all exhibiting statistical significance, except for the forearm flap (Fig. 2).

## Discussion

In the CST from 2017 to 2021, it was difficult for an instructor to point out an unstained cadaver flap nutrient artery and its perforators to trainees. Although highly experienced instructors performing the demonstration can identify blood vessels and elevate the skin flap relatively easily in unstained cadavers, this is often difficult for inexperienced trainees.

In this study, water-soluble staining solutions were used on cadavers immediately prior to the lecturer’s demonstration; SSS-fixed and urea-treated cadavers stained well, probably because of the higher water content and softness of the cadavers, which resulted in better vascular compliance. Except for the medial plantar flap, in which a nutrient artery was thought to be occluded because of atherosclerosis, the staining was clear and highly visible. Because most of the cadavers were elderly, such peripheral arterial occlusions were considered unavoidable.

The participants gave a high rating for the staining of blood arteries, and more than 90% of them requested staining of cadaver nutrient arteries for the CST. In addition, the increase in the trainees’ skill acquisition levels for flap elevation before and after CST was greater when a cadaver with stained nutrients and perforator arteries was used. These results may be attributed to the improved surgical image, which led to the easy identification of blood vessels by looking at a demonstration with stained arteries.

However, this study had some limitations. First, its conclusions were based on a limited number of participants and cadavers, which may not be representative of a larger population of surgical trainees and cadaveric specimens. Second, this was a single-center study, and the findings may not be generalizable to other settings with different training environments. Third, dye leakage into the surrounding tissues can affect the clarity and precision of the anatomical structures being studied. Finally, the study lacked a long-term follow-up to assess whether the improved skills were retained over time and translated into clinical practice. In addition, this method may make it difficult to observe structures other than blood vessels because of dye leakage into the tissue surrounding the artery. During digital artery flap elevation, the dye seeps into the surrounding area after dissection of the artery. Proper ligation of the blood vessels and the use of microclips and other devices should be considered. Additionally, this method requires a notable amount of staining time. Staining all cadavers is time-consuming and difficult to perform in advance. If staining is conducted too early, the perivascular tissue may be stained because of the water-soluble nature of the stain. Thus, it is necessary to investigate whether staining with non-water-soluble dyes, such as latex or silicone, can be achieved with a similarly fixed cadaver.

Various methods, such as paint, gelatin, latex, silicone, and adalurite, have been used to stain blood vessels in freshly frozen cadavers and formalin-fixed cadaver upper extremities. Although acrylic paint can depict microvessels, staining of nonvascular tissues is problematic because of leakage. Relatively fine blood vessels were also observed when latex was used. Silicon provides better delineation than latex in 24–48 h; however, its high cost and toxicity are disadvantages. Adalurite is inflexible, and its stiffness is problematic (Doomernik et al. 2016). Hupkens et al. (2010) reported that a 2-day freezing period after paint injection reduced leakage during specimen preparation of the perforating branch of the anterolateral femoral cutaneous flap. Maga et al. (2013) reported a 2-week formalin fixation period after paint injection in a study on cerebral vascular specimen preparation methods. Pérez-Cruz et al. (2024) reported that a 24-h vascular washing process is essential, and meticulous leak prevention is crucial before dye injection. Tamai (2024) successfully injected latex into fresh cadavers and stained the microvasculature in the fingers. However, these studies primarily focused on the preparation of stained specimens. In the context of surgical training, where tissue flexibility is essential, treatments such as drying and formalin fixation that induce hardening may present certain drawbacks. Ideally, staining fresh cadavers would be preferable; however, obtaining a sufficient number of fresh cadavers for training purposes can sometimes be challenging. In our seminar, we use 10 cadavers per session, and while achieving clear vascular staining is essential, there is also a need to develop methods to stain the vasculature of multiple cadavers more efficiently. Conducting staining in a time-effective manner is crucial for accommodating the demands of larger training sessions.

A few studies have been conducted on plastic surgery training. Wong et al. (2023) reported that fresh cadavers injected with latex and fixed at a low temperature for 2–5 days stained well, down to the perforating branches of the skin flap. Wannatoop et al. (2022) reported that a perfusion model (perfused cadavers) improved the reality of CST trauma and resulted in higher participant satisfaction than conventional CST. However, the perfusion model may be more difficult for inexperienced participants, because the dye will continue to leak if the vessels are improperly treated.

The strength of our study is that we quantitatively evaluated the improvement in the skills of the participants, specifically examining the effects of donor staining on skill enhancement. Although staining all donor specimens is expected to be time- and manpower-intensive, this study showed that staining the instructor’s donor cadaver alone improved the participants’ proficiency. Further studies are required to determine whether staining of all donor cadavers should be performed, considering the level of training and cost involved. Therefore, more efficient dyes and staining methods are required.

## Conclusions

Visualization of blood vessels in instructor donor cadavers improved trainees’ skills. These results suggest that SSS-fixed and urea-treated cadaveric vascular staining models may be useful as training models for skin flap elevation.

## Data Availability

The data that support the findings of this study are available from the corresponding author upon reasonable request.
